# Psychometric testing of the Norwegian version of the Simulation Design Scale, the Educational Practices Questionnaire and the Student Satisfaction and Self-Confidence in Learning Scale in nursing education

**DOI:** 10.1016/j.ijnsa.2020.100012

**Published:** 2020-10-25

**Authors:** Inger Åse Reierson, Leiv Sandvik, Hilde Solli, Thor Arne Haukedal, Sissel Eikeland Husebø

**Affiliations:** a Department of Nursing and Health Sciences, Faculty of Health and Social Sciences, University of South-Eastern Norway, Pb 235, 3603 Kongsberg, Norway; b Department of Quality and Health Technology, Faculty of Health Sciences, University of Stavanger, Pb 8600, 4036 Stavanger, Norway

**Keywords:** Evaluation tool, Nursing education, Psychometric testing, Simulation-based learning

## Abstract

**Introduction:**

Simulation-based learning is a well-established technique in nursing education. However, there is a need for reliable and validated evaluation tools across both national boundaries and cultural conditions. Such evaluation tools may contribute in identifying areas for improvement in simulation-based learning from the nursing students’ perspective.

**Objectives:**

The aim of this study was to test three widely used American questionnaires – the *Simulation Design Scale,* the *Educational Practices Questionnaire,* and the *Student Satisfaction and Self-Confidence in Learning Scale,* for psychometric properties among Norwegian undergraduate nursing students.

**Methods:**

A descriptive cross-sectional study was conducted at a university simulation center in southern part of Norway. A total of 105 undergraduate nursing students participated, giving a response rate of 77%. An exploratory factor analysis was used to examine construct validity. Cronbach's alpha was applied in order to establish the questionnaires’ internal consistency.

**Results:**

The exploratory factor analyses displayed the same number of extracted factors as the number of subscales in each of the original American questionnaires. However, the item-factor structure differed from the original item-subscales. The Cronbach's alpha was > 0.7 for all three questionnaires, indicating acceptable internal consistency.

**Conclusion:**

Psychometric testing of the Norwegian versions of the three questionnaires, the *Simulation Design Scale,* the *Educational Practices Questionnaire,* and the *Student Satisfaction and Self-Confidence in Learning Scale*, could be used as valid instruments for nursing students to evaluate important aspects of simulation-based learning. This also makes it easier to compare evaluation results of SBL across languages and cultural boundaries. However, to confirm the construct validity of the factors extracted in this study, further multi-site studies are needed to perform a confirmatory factor analysis in a new, large sample.

## Contribution of the paper

What is already known about the topic?

• Simulation-based learning is a widely used technique in nursing education.
• There is a lack of valid and reliable tools to evaluate this resource intensive technique.

What this paper adds

• This study demonstrates that the psychometric testing of SDS, EPQ and SCLS in a Norwegian context maintains internal consistency.
• The number of extracted factors corresponds to the subscales in the original questionnaires.
• Item-factor structure should be adjusted compared to the original American version.

## 1 Introduction

Simulation-based learning (SBL) has been used as an educational method in undergraduate nursing education for more than a century, and research since the 1990s has documented its increasingly extensive use (Aebersold, 2018). This method provides an opportunity to explore nursing scenarios and train students in a risk-free environment, preparing them for their clinical practice (Cant and Cooper, 2017). Simulation-based learning has been found to improve satisfaction, self-confidence and self-efficacy in learning situations (Haddeland et al., 2018; Warren et al., 2016) and also to improve important nursing attributes such as clinical judgment, problem solving, critical thinking, psychomotoric skills and theoretical knowledge (Al Sabei and Lasater, 2016; Haukedal et al., 2018; Jeppesen et al., 2017). In view of the challenges related to the scarcity of clinical placements and concern for patient safety, SBL has increasingly been highlighted in a global context, as preclinical preparation or as a replacement for parts of clinical practice, depending on different countries’ statutory education requirements (Aebersold, 2018; Hayden et al., 2014).

Simulation is defined as “a technique that creates a situation or environment to allow persons to experience a representation of a real event for the purpose of practice, learning, evaluation, testing, or to gain understanding of systems or human actions” (Lioce et al., 2020, p. 44). Simulation attempts to achieve a level of fidelity sufficient to convince users that they are engaged in situations that mimic real life. Fidelity is described as the precision of reproduction of real life and is qualified as low, medium, or high depending on the degree to which a simulated experience approaches reality; thus, as fidelity increases, so does realism (INACSL, 2016). The level of fidelity is determined by the environment, the tools and resources used, and factors associated with the participants (Almeida et al., 2018; Cant and Cooper, 2017; INACSL, 2016). However, SBL often requires substantial financial investment, in both equipment and personnel (Lovett et al., 2016). Many stakeholders, including educators, university and hospital administrators, and clinical staff, have a vested interest in the use of simulation as a valid and reliable educational technique (Adamson et al., 2013; Franklin et al., 2014). Robust evaluation using rigorous and valid methods is essential to reassure stakeholders of the value of simulation within healthcare.

To enhance the quality of SBL, the National League of Nursing (NLN)/Jeffries simulation framework was developed to support facilitators in implementing simulation in nursing education (Jeffries, 2005). The framework specifies features to include in three main areas in simulation development: *simulation design characteristics* (objectives, fidelity, problems solving, student support and debriefing), *educational practices* (active learning, feedback, student/faculty interaction, collaboration, high expectations, diverse learning and time on task) and *outcomes* (learning, knowledge, skill performance, learner satisfaction, critical thinking and self-confidence) (Jeffries and Rogers, 2012).

To obtain knowledge from evidence-based evaluations, it is important to use validated tools (Adamson et al., 2013; Cant and Cooper, 2017). A review of studies on SBL in undergraduate nursing education revealed a lack of valid and reliable evaluation tools to assess SBL (Doolen et al., 2016). Franklin et al. (2014) found that in the majority of non-experimental studies, non-validated self-efficacy scales were utilized. This also applies for other self-reported outcomes such as confidence, competence and satisfaction (Cant and Cooper, 2017). Hence, there is a need for more robust questionnaires to evaluate SBL in nursing education (Adamson et al., 2013; Almeida et al., 2018; Kardong-Edgren et al., 2010).

Various evaluation questionnaires have been developed for application in SBL (Adamson et al., 2013; Kardong-Edgren et al., 2010). Of these, the three most widely used, self-reported questionnaires have been developed by the NLN: the *Simulation Design Scale* (SDS), the Ed*ucational Practices Questionnaire* (EPQ), and the *Student Satisfaction and Self-Confidence in Learning Scale* (SCLS) (Adamson et al., 2013; Franklin et al., 2014; Jeffries and Rogers, 2012; Kardong-Edgren et al., 2010; National League of Nursing, 2020a). These questionnaires were developed to evaluate SBL in undergraduate nursing education (Jeffries, 2005; Jeffries and Rogers, 2012). They were theoretically based on the NLN/Jeffries simulation framework (Jeffries and Rizzolo, 2006; Jeffries and Rogers, 2012), which underscores the three specific features in SBL: simulation design, implementation of educational practices and outcomes. Each of these features corresponds to the above-mentioned student self-reported measurement instruments: SDS, EPQ, and SCLS (Jeffries and Rogers, 2012). In SDS and EPQ, respondents evaluate SBL in two ways: namely, they assess the presence of (PO) key aspects and the importance of (IO) said aspects. The three questionnaires are designed to evaluate the affective domain (Kardong-Edgren et al., 2010) and focus on Kirkpatrick's level 1; Kirkpatrick's model of training evaluation criteria is a widely used framework that consists of four criteria and is often used in simulation research (Kirkpatrick, 1996; Aebersold, 2018). Reaction criteria (Level 1) represent the extent to which students enjoy the training and/or find it useful. Using validated tools to evaluate student feedback on their perspectives regarding key features of simulation is essential to improving and adjusting simulation as a learning method in nursing education.

Previous research shows that evidence of psychometric testing of the three questionnaires is scarce (Almeida et al., 2015; Chan et al., 2015; Franklin et al., 2014; Tosterud et al., 2014; Unver et al., 2017). Franklin et al. (2014) were the first to carry out psychometric testing of SDS-PO, EPQ-PO, and SCLS among novice nurses in a pre-licensure baccalaureate nursing program at a university in the USA. The study found SDS-PO and SCLS to be reliable and valid instruments; EPQ-PO was found to be reliable, but a stable factor solution was not supported for this scale (Franklin et al., 2014). Unver et al. (2017) found the SDS-PO, EPQ-PO, and SCLS questionnaires to have acceptable psychometric properties in undergraduate nursing education in a Turkish context. Chan et al. (2015) tested SCLS for psychometric properties in a Chinese context, among practicing nurses in an advanced life-support course and found the questionnaire to be a reliable and valid tool. Almeida et al. (2015) translated the SCLS questionnaire into Portuguese and tested it for psychometric properties in both Portuguese and Brazilian contexts among degree level and non-degree level nurses. They found the questionnaire to have acceptable psychometric properties. All three questionnaires – SDS, EPQ and SCSL – have been translated into Norwegian (Tosterud, 2015), but only SCLS has been tested for psychometric properties, showing internal consistency but no stable factor solution (Tosterud et al., 2014). Further psychometric evaluation of SDS, EPQ and SCLS for a Norwegian context are needed.

### 1.1 Aim

The aim of the present study was to test three questionnaires: – the *Simulation Design Scale* (SDS), the *Educational Practices Questionnaire* (EPQ), and the *Student Satisfaction and Self-Confidence in Learning Scale* (SCLS) for psychometric properties among Norwegian undergraduate nursing students after conducting a compulsory SBL course. The surveys were conducted as part of a larger study on the evaluation of scenario simulation from the perspective of nursing students.

## 2 Methods

### 2.1 Study design, sample and setting

This study was designed as a descriptive cross-sectional study (Polit and Beck, 2018). A convenience sample was chosen since the respondents were recruited from a compulsory SBL course (Patton, 2015). In total, 137 nursing students (83 full time students and 54 part-time students) in the second year of an undergraduate program in nursing at a university in southern Norway were invited to participate in the study. The simulation sessions took place in the simulation center at the university. Nursing students attending the compulsory SBL course were divided into a total of 15 groups of 7–11 students. Each student participated in hands-on simulation 1-2 times during the six scenarios which simulated acutely deteriorated patient situations. Three advanced simulators were used in the simulations (one Laerdal SimMan 3G® and two Laerdal ALS®). Learning objectives in all scenarios included assessing and acting in relation to situations requiring the *Airway, Breathing, Circulation, Disability, Exposure (ABCDE)* approach (Thim et al., 2012), *Secure Communication* and *Leadership*. The simulation sessions consisted of briefing, simulation and debriefing. In designing the entire simulation session, the International Nursing Association for Clinical Simulation and Learning (INACSL) standards for best practice (INACSL, 2016) were applied.

### 2.2 Ethics

The Norwegian Center for Research Data (NSD no: 56123) and the Dean of the university approved this study. The study was conducted according to the Declaration of Helsinki and ethical guidelines for research (World Medical Association, 2001). Safeguards were put in place to ensure the confidentiality of the participating students. Potential participants received a self-administered questionnaire together with written information about the study, its significance and associated ethical issues; this information was also given verbally to the participants prior to the day on which data collection took place. It was emphasized that participation was voluntary, that they could withdraw from the study at any time and that the decision to participate or decline to, would not influence the participants’ studies. This information was crucial because the first, second and third author were educators at the university where the study was performed. The second and third authors, who gathered the data, were also involved in a subsequent practical nursing skills examination of the students; however, this examination had different learning objectives compared to the scenario simulation activities. The first author took part in data collection in the sample of the fulltime students.

All participants signed a letter of consent before they were enrolled in the study. All data were collected anonymously and were coded to maintain confidentiality.

### 2.3 Questionnaires

The American version of each of the three questionnaires, SDS, EPQ and SCLS, is permitted for use in research (National League of Nursing, 2020b). Permission to use the Norwegian version of the questionnaires was obtained from Tosterud. The three questionnaires are presented in the following.

#### 2.3.1 Simulation Design Scale

The SDS questionnaire is a 20-item, self-report questionnaire designed to evaluate five central design dimensions in SBL (National League of Nursing, 2020a). The scale's two parts, PO and IO, each consist of five specific dimensions: Objectives and Information (five items), Support (four items), Problem Solving (five items), Feedback/Guided Reflection (four items) and Fidelity/Realism (two items) (Jeffries and Rizzolo, 2006). In SDS-PO, the responses are graded from 1 (Strongly disagree) to 5 (Strongly agree) on a five-point Likert scale. In SDS-IO, the responses are graded from 1 (Not important) to 5 (Very Important) on a five-point Likert scale. The American questionnaire's Cronbach's alpha was 0.92 for SDS-PO and 0.96 for SDS-IO (National League of Nursing, 2020a).

#### 2.3.2 Educational Practices Questionnaire

The EPQ questionnaire is a 16-item, self-reported questionnaire that was designed to evaluate educational practices in simulation (National League of Nursing, 2020a). The scale's two parts, PO and IO, each consist of four dimensions: Active Learning (ten items), Collaboration (two items), Diverse Ways of Learning (two items) and High Expectations (two items) (Jeffries and Rizzolo, 2006). In EPQ-PO, the responses are graded from 1 (Strongly disagree) to 5 (Strongly agree) on a five-point Likert scale. In EPQ-IO, the responses are graded from 1 (Not important) to 5 (Very Important) on a five-point Likert scale. The American questionnaire's Cronbach's alpha was 0.86 for EPQ-PO and 0.91 for EPQ-IO (National League of Nursing, 2020a).

#### 2.3.3 Student Satisfaction and Self-Confidence in Learning Scale

The SCLS questionnaire is a 13-item, self-reported questionnaire designed to assess satisfaction with instruction and self-confidence in SBL (Jeffries and Rizzolo, 2006). The scale consists of two dimensions: Satisfaction with Current Learning (five items) and Self-Confidence in Learning (eight items). In SCLS, the responses are graded from 1 (Strongly disagree) to 5 (Strongly agree) on a five-point Likert scale. The American questionnaire's Cronbach's alpha was 0.94 for the Satisfaction subscale and 0.87 for the Self-Confidence subscale (National League of Nursing, 2020a).

### 2.4 Data collection

Data collection took place in December 2017 (full-time students) and May 2018 (part-time students), immediately after the nursing students had completed all simulation scenario activities in the compulsory SBL course in their second year. The questionnaires were distributed to the students in a paper–pencil version.

### 2.5 Data analysis

The data were analyzed using Statistical Package for the Social Sciences (IBM Corp. 2019). Psychometric testing was conducted on the SDS, EPQ and SCLS. SDS-PO, SDS-IO, EPQ-PO, and EPQ-IO were tested separately. Prior to analysis, the data were cleaned as described by Pallant (2016).

#### 2.5.1 Reliability

Descriptive analysis was conducted to determine items’ response mean, standard deviation, skewness, kurtosis and item total correlations (Pallant, 2016). Skewness less than −1 and larger than 1 meant the response distribution was considered highly skewed to the right or to the left, respectively (Tabachnick and Fidell, 2019). Positive kurtosis represents a peaked distribution of values with negative kurtosis representing a flatter than normal distribution of values (Tabachnick and Fidell, 2019). An item total correlation coefficient between 0.30 and 0.70 was considered significant (Tabachnick and Fidell, 2019). To calculate the internal consistency of the questionnaires, a Cronbach's Alpha was chosen with values > 0.70 regarded as acceptable and > 0.80 as preferable (Pallant, 2016). Internal consistency was computed for each final extracted factor in each questionnaire (Pett et al., 2003).

#### 2.5.2 Validity

An exploratory factor analysis (EFA) was conducted to uncover the factor structure of the three questionnaires SDS, EPQ and SCLS, which included separately testing SDS-PO, SDS-IO, EPQ-PO, and EPQ-IO. EFA was chosen because this was the first time all three translated questionnaires were tested in the same study in a Norwegian context and hence no a priori factor structure hypothesis had been presumed (Pallant, 2016; Tabachnick and Fidell, 2019). The exclude cases pairwise option in SPSS was chosen to handle missing data (Pallant, 2016). Prior to performing EFA, we assessed the suitability of our data for factor analysis. Decision on sample size is highlighted in EFA. A larger sample size is generally viewed as creating more stable estimates of factor loadings; however, there is no consensus on how large the sample size should be to perform EFA (Hogarty et al., 2005). Hair et al. (2014) recommend having a sample size larger than 100 and at least five times as many observations as the number of variables. Additionally, de Winter et al. (2009) found that EFA can yield reliable results for sample sizes well below 50. Based on Hogarty et al. (2005), Hair et al. (2014), de Winter et al. (2009) and the appraisal of one of the authors (LS), a statistician, the sample size in this study was considered large enough to perform an EFA. Bartlett's test of sphericity was conducted to test the overall significance of differences in the correlation matrix, with a value of *p*< 0.05 for EFA considered to be appropriate (Pallant, 2016; Tabachnick and Fidell, 2019). Furthermore, the Kaiser–Meyer–Olkin (KMO) test was performed to measure the sample adequacy. Within the KMO range of 0 to 1, a value of 0.60 or above was required in order to conduct an EFA (Tabachnick and Fidell, 2019). A principal component analysis (PCA) was conducted to reveal the number of components with eigenvalues exceeding 1.0 (Pallant, 2016; Polit and Beck, 2018). Inspection of the scree plot was used to further confirm the number of components (Pallant, 2016; Tabachnick and Fidell, 2019). To aid the interpretation of the components, an oblique rotation produced by the direct oblimin was used to check how the items correlated with the components and to ascertain the degree of correlation between the extracted factors. Factor loadings between 0.30 and 0.70 were considered acceptable (Hair et al. 2014; Pallant, 2016). After the initial EFA, the research team discussed the appropriateness of the item-factors content (Tabachnick and Fidell, 2019; Watkins, 2018); this was done for each of the three questionnaires. When PCA and scree plot analysis revealed the same number of components, these numbers were retained. For SDS-IO and EPQ-PO, the numbers of suggested components from PCA and scree plot analysis differed. Based on reflections in the research group regarding the meaningfulness of item-factor structure and literature supporting the idea that scree plots may be more precise than eigenvalues (Tabachnick and Fidell, 2019), a second EFA was performed for SDS-IO and EPQ-PO with a fixed component solution.

## 3 Results

### 3.1 Characteristics of the sample

A total of 105 (77% of those invited to participate) nursing students responded to the questionnaires. Of these, 71 (68%) were full-time students and 34 (32%) were part-time. Female nursing students constituted the majority of respondents (84%). The mean age of the sample was 26 years (SD=7.1). Most of the nursing students (91%) had no previous experience with SBL using technologically advanced simulators. The mean score of the number of simulations was 1.7 for students with prior simulation experience.

### 3.2 Psychometric analysis of Simulation Design Scale

#### 3.2.1 Reliability

The item analysis of the SDS-PO and SDS-IO scales is presented in Table 1. The item response frequencies show that most respondents answered either *agree* or *strongly agree* for SDS-PO and either *important* or *very important* for SDS-IO. For SDS-PO skewness was less than −1 in 19 of 20 items. Kurtosis values were above zero in 19 of 20 items. Inter-item correlations were all above 0.30 except for item D19 (0.24) and item D20 (0.24). The low scores for items D19 and D20 indicate that these might be removed from the scale. For SDS-IO, skewness was less than −1 in 17 of 20 items. Kurtosis values were above zero in 19 of 20 items. Inter-item correlations were all above 0.30 except for item D19 (0.23), which indicates that item D19 might be removed. Cronbach's alpha value was 0.88 for total SDS-PO and 0.91 for total SDS-IO. For the SDS-PO subscales (*Objectives and Information, Support, Problem Solving, Feedback/Guided Reflection and Fidelity (Realism)*), the Cronbach's alpha values were 0.76, 0.89, 0.69, 0.74 and 0.78 respectively. For the SDS-IO subscales (*Objectives and Information, Support, Problem Solving, Feedback/Guided Reflection and Fidelity (Realism)*), the Cronbach's alpha values were 0.77, 0.92, 0.72, 0.80 and 0.73 respectively.

**Table 1:** Response option frequency for SDS.

Item	SDS-PO							SDS-IO						
	SD %	D%	UN %	A %	SA %	Mean ± SD	Item Total Correlation	NI %	SI %	N %	I %	VI %	Mean ± SD	Item total correlation
D1. There was enough information provided for direction and encouragement.	2.9	8.6	18.1	39.0	29.5	3.85±1.042	0.44		1.9	3.8	25.7	64.8	4.59±0.666	0.64
D2. I clearly understood the purpose and objectives of the simulation.		2.9	5.7	29.5	60.0	4.50±0.739	0.64	1.0		2.9	28.6	61.9	4.60±0.653	0.60
D3. The simulation provided information in a clear matter for me to problem-solve.	1.0	5.7	20.0	37.1	33.3	3.95±1.013	0.56			4.8	27.6	62.9	4.61±0.584	0.57
D4. There was enough information provided to me during the simulation.		2.9	18.1	39.0	38.1	4.15±0.821	0.58			8.6	26.7	61.0	4.54±0.656	0.61
D5. The cues were appropriate and geared to promote my understanding.		2.9	15.2	36.2	39.0	4.03±1.147	0.55	1.0	1.0	8.6	26.7	57.1	4.46±0.787	0.57
D6. Support was offered in a timely manner.	1.0	2.9	13.3	36.2	41.0	4.04±1.179	0.63	1.0	1.0	4.8	30.5	59.0	4.51±0.730	0.63
D7. My need for help was recognized.	1.0	3.8	11.4	36.2	41.0	4.00±1.252	0.64	1.0	1.0	5.7	28.6	59.0	4.51±0.745	0.65
D8. I felt supported by the teacher's assistance during the simulation.	1.9	1.9	9.5	30.5	50.5	4.17±1.213	0.60	1.9		3.8	24.8	65.7	4.58±0.752	0.74
D9. I was supported in the learning process.			5.7	35.2	53.3	4.37±0.974	0.63	1.0		4.8	23.8	65.7	4.61±0.680	0.66
D10. Independent problem-solving was facilitated.			12.4	22.9	59.0	4.28±1.194	0.32	1.0		4.8	26.7	60.0	4.57±0.691	0.52
D11. I was encouraged to explore all possibilities of the simulation.		5.7	30.5	26.7	32.4	3.79±1.143	0.61		2.9	16.2.	29.5	46.7	4.26±0.848	0.61
D12. The simulation was designed for my specific level of knowledge and skills.		2.9	7.6	51.4	36.2	4.23±0.717	0.48			7.6	29.5	59.0	4.53±0.641	0.56
D13. The simulation allowed me the opportunity to prioritize nursing assessments and care.			4.8	18.1	75.2	4.72±0.550	0.46			2.9	22.9	70.5	4.70±0.520	0.52
D14. The simulation provided me an opportunity to goal set for my patient.	1.0	4.8	21.0	41.0	28.6	3.87±1.045	0.50	2.9	1.0	11.4	39.0	41.0	4.20±0.910	0.49
D15. The feedback provided was constructive.			1.9	17.1	79.0	4.79±0.457	0.51				18.1	78.1	4.81±0.393	0.55
D16. Feedback was provided in a timely manner.			2.9	19.0	76.2	4.75±0.499	0.50			1.9	21.0	73.3	4.74±0.483	0.61
D17. The simulation allowed me to analyze my own behavior and actions.			1.9	15.2	80.0	4.77±0.581	0.32	1.0		1.9	16.2	77.1	4.75±0.590	0.48
D18. There was an opportunity after the simulation to obtain guidance/feedback from the teacher.		1.9	6.7	11.4	78.1	4.69±0.686	0.49			1.9	14.3	80.0	4.81±0.441	0.65
D19. The scenario resembled a real-life situation.			1.0	8.6	88.6	4.89±0.340	0.24			1.9	8.6	84.8	4.87±0.393	0.23
D20. Real life factors situations and variables were built into the simulation scenario.			1.0	18.1	79.0	4.80±0.428	0.24				11.4	83.8	4.88±0.327	0.39

SD = Strongly Disagree. D = Disagree. UN = Undecided. A = Agree. SA = Strongly Agree. NI= Not Important. SI = Somewhat Important. N = Neutral. I = Important. VI = Very Important.

#### 3.2.2 Validity

Bartlett's test of sphericity and KMO revealed sample adequacy for conducting factor analyses for both SDS-PO and SDS-IO. The Bartlett's test showed significance for SDS-PO (χ2 = 987.91, *P* < .001) and SDS-IO (χ2=1087.33, *P* < .001). The KMO showed 0.80 for SDS-PO and 0.83 for SDS-IO. For SDS-PO, the initial EFA resulted in a five-component model that explained 66.8% of the variance. The scree plot analysis supported this five-component solution when using the inspection method described by Field (2009) (Supplementary file 1). For SDS-IO, the initial EFA resulted in a four-component model that explained 64.3% of the variance. However, an inspection of the SDS-IO scree plot supported a five-component solution (Supplementary file 1). An oblique rotation was performed for both SDS-PO and SDS-IO. Since scree plots are often viewed as more precise than eigenvalues in estimating the number of components (Tabachnick and Fidell, 2019), the research group decided to conduct a second EFA for the SDS-IO with a fixed five component solution. In the second EFA for the SDS-IO, Bartlett's test of sphericity was significant (χ2 = 1087.33, *P* < .001) and KMO was 0.83. The five-component solution explained 69.0% of the variance. An oblique rotation was performed. In order to ascertain the meaningfulness of item-component structure, the research team then inspected the five-component solution for both SDS-PO and SDS-IO in terms of the item-component structure. Collectively, the researchers were well experienced in the subject matter of SBL and applied their competency when interpreting the analysis, which supported a five-factor solution for both SDS-PO and SDS-IO. Such reflection is underscored in the literature (Kentaro and Yuan, 2010; Osborne, 2014). Factor loadings and communalities are presented in Table 2. Table 2 also shows that even though the number of factors extracted is the same for SDS-PO and SDS-IO, the item-factor structure varies. The pattern matrix and structure matrix for SDS-PO and SDS-IO are presented in Supplementary file 2.

**Table 2:** Exploratory factor analysis for SDS-PO, SDS-IO, EPQ-PO, EPQ-IO, SCLS; factor loadings and communalities (h^2^) Factors’ Cronbach's alpha.

Subscales with items	SDS-PO						SDS-IO					
	Factor loadings					h^2^	Factor loadings					h^2^
	Factor 1	Factor 2	Factor 3	Factor 4	Factor 5		Factor 1	Factor 2	Factor 3	Factor 4	Factor 5	
**Objectives and information**												
D1	0.87					0.66				−0.64		0.61
D2	0.76					0.74	0.73					0.65
D3	0.57					0.58	0.75					0.66
D4	0.57					0.59	0.50					0.53
D5		−0.74				0.56		−0.66				0.63
**Support**												
D6		−0.89				0.76		−0.89				0.78
D7		−0.83				0.73		−0.86				0.80
D8		−0.86				0.71		−0.82				0.77
D9		−0.83				O.74		−0.91				0.83
**Problem Solving**												
D10				0.83		0.77	0.56					0.45
D11		−0.42				0.58	0.42					0.65
D12					0.67	0.61					−0.70	0.73
D13					0.59	0.57					−0.83	0.77
D14				0.62		0.62				−0.62		0.66
**Feedback/Guided Reflection**												
D15	0.45					0.69				−0.61		0.69
D16			0.48			0.68				−0.77		0.71
D17					0.88	0.72					-0.82	0.72
D18	0.60					0.54				−0.66		0.81
**Fidelity (Realism)**												
D19			0.86			0.74			0.78			0.69
D20			0.86			0.75			0.69			0.67
**Cronbach's alpha**	0.82	0.89	0.69	0.71	0.72		0.78	0.91	0.73	0.76	0.72	


Subscales with items	EPQ-PO						EPQ-IO					
	Factor loadings				h^2^		Factor loadings				h^2^	
	Factor 1	Factor 2	Factor 3	Factor 4			Factor 1	Factor 2	Factor 3	Factor 4		
**Active Learning**												
E1		−0.53			0.40		0.46				0.22	
E2			0.79		0.63			0.77			0.70	
E3			0.89		0.78			0.81			0.69	
E4	0.52				0.53		0.66				0.55	
E5			0.57		0.58					−0.69	0.74	
E6	0.78				0.54		0.58				0.59	
E7	0.69				0.58		0.80				0.63	
E8	0.57				0.50		0.53				0.57	
E9	0.71				0.56					−0.57	0.60	
E10				−0.40	0.49					−0.54	0.60	
**Collaboration**												
E11		−0.89			0.73				−0.75		0.71	
E12		−0.76			0.65				−0.82		0.76	
**Diverse Ways of Learning**												
E13	0.41				0.37					−0.80	0.77	
E14	0.49				0.35					−0.72	0.76	
**High Expectations**												
E15				−0.82	0.74		0.72				0.63	
E16				−0.74	0.65		0.54				0.56	
**Cronbach's alpha**	0.78	0.58	0.66	0.71			0.79	0.71	0.79	0.83		


Subscales with items	SCLS											
	Factor loadings		h^2^									
	Factor 1	Factor 2										
**Satisfaction with Current Learning**												
S1	0.74		0.53									
S2	0.78		0.57									
S3	0.49		0.56									
S4	0.72		0.60									
S5		−0.64	0.67									
**Self-confidence in Learning**												
S6		−0.69	0.53									
S7		−0.71	0.45									
S8	0.53		0.49									
S9	0.80		0.67									
S10	0.64		0.36									
S11		−0.80	0.59									
S12		−0.80	0.69									
**Cronbach's alpha**	0.83	0.82										

#### 3.2.3 Additional assessment of reliability

After the research team concluded on the number of factors to retain, Cronbach's alpha was conducted for the five factors extracted in SDS-PO and SDS-IO, see Table 2.

### 3.3 Psychometric analysis of Educational Practices Questionnaire

#### 3.3.1 Reliability

The item analysis of the EPQ-PO and EPQ-IO is presented in Table 3. The item response frequencies show that most respondents answered *agree* or *strongly agree* for EPQ-PO and either *important* or *very important* for EPQ-IO. For EPQ-PO, skewness was less than -1 in 15 of 16 items. Kurtosis values were above zero in 15 of 16 items. Inter-item correlations were all above 0.30 except for item E3 (0.29), which indicates that this item might be removed. For EPQ-IO, skewness was less than -1 in 14 of 16 items. Kurtosis values for all 16 items were above zero. Inter-item correlations were all above 0.30. Cronbach's alpha was 0.82 for EPQ-PO and 0.88 for EPQ-IO. For the EPQ-PO subscales (*Active Learning, Collaboration, Diverse Ways of Learning and High Expectations*), the Cronbach's alphas were 0.77, 0.74, 0.63 and 0.72 respectively and for the EPQ-IO subscales (*Active Learning, Collaboration, Diverse Ways of Learning and High Expectations*), 0.80, 0.79, 0.84 and 0.76 respectively.

**Table 3:** Response option frequency for EPQ.

Item	EPQ-PO							EPQ-IO						
	SD %	D %	UN %	A %	SA %	Mean ± SD	Item-total correlation	NI %	SI %	N %	I %	VI %	Mean ± SD	Item-total correlation
E1. I had the opportunity to discuss the ideas and concepts with the teacher and other students.	2.9	1.9	8.6	23.8	61.9	4.38±1.055	0.34	1.0	2.9	8.6	28.6	55.2	4.40±0.849	0.32
E2. I actively participated in the debriefing session after the simulation.	1.0	2.9	5.7	31.4	58.1	4.44±0.810	0.32	1.9		3.8	37.1	54.3	4.46±0.753	0.47
E3. I had the opportunity to put more thought into my comments during the debriefing session.	1.0	1.0	4.8	31.4	60.0	4.51±0.726	0.29		1.9	6.7	36.2	52.4	4.43±0.711	0.38
E4. There were enough opportunities to find out if I clearly understand the material.		1.9	15.2	41.0	41.0	4.22±0.775	0.55		2.9	10.5	30.5	52.4	4.38±0.798	0.56
E5. I learned from the comments made by the teacher before, during or after the simulation.			1.0	27.6	70.5	4.70±0.480	0.49			1.0	26.7	68.6	4.70±0.481	0.59
E6. I received cues during the simulation in a timely manner.	1.9	3.8	23.8	38.1	30.5	3.89±1.014	0.38	1.9	1.0	13.3	39.0	41.0	4.21±0.864	0.37
E7. I had the chance to discuss the simulation objectives with my teacher.		2.9	20.0	31.4	41.9	4.13±0.951	0.53	1.0	4.8	14.3	35.2	39.0	4.13±0.922	0.56
E8. I had the opportunity to discuss ideas and concepts with my instructor.		1.9	8.6	32.4	54.3	4.39±0.854	0.55		1.0	11.4	31.4	51.4	4.40±0.739	0.68
E9. The instructor was able to respond to the individual needs of learners,	1.0	7.6	21.0	28.6	39.0	3.92±1.146	0.55		1.0	6.7	36.2	52.4	4.46±0.671	0.62
E10. Using simulation activities made my learning time more productive		1.0	2.9	14.3	81.0	4.77±0.544	0.49			1.9	20.0	74.3	4.75±0.478	0.53
E11. I had the chance to work with my peers.	1.9		1.9	18.1	76.2	4.65±0.833	0.32		1.0	5.7	21.9	68.6	4.63±0.644	0.54
E12. During the simulation, my peers and I had to work on the clinical situation together.	1.0	1.0	3.8	17.1	76.2	4.68±0.686	0.36		2.9	2.9	25.7	65.7	4.59±0.694	0.49
E13. The simulation offered a variety of ways in which to learn the material.	1.0			21.0	77.1	4.75±0.553	0.44		1.0	1.9	22.9	71.4	4.70±0.559	0.63
E14. This simulation offered a variety of ways of assessing my learning.			6.7	21.0	71.4	4.65±0.604	0.44	1.0		4.8	21.9	69.5	4.64±0.672	0.60
E15. The objectives were clear and easy to understand.		4.8	6.7	37.1	50.5	4.35±0.810	0.49		1.0	8.6	31.4	56.2	4.47±0.699	0.63
E16. My instructor communicated the goals and expectations to accomplish.		1.0	10.5	29.5	58.1	4.46±0.723	0.45		1.0	6.7	32.4	57.1	4.50±0.671	0.60

SD = Strongly Disagree. D = Disagree. UN = Undecided. A = Agree. SA = Strongly Agree. NI= Not Important. SI = Somewhat Important. N = Neutral. I = Important. VI = Very Important.

#### 3.3.2 Validity

Bartlett's tests of sphericity and KMO revealed that the sample met the criteria for conducting factor analyses for both EPQ-PO and EPQ-IO. Bartlett's test was significant for EPQ-PO (χ2 = 478.64, *P* <.001) and EPQ-IO (χ2 = 673.62, *P* <.001). The KMOs for EPQ-PO and EPQ-IO were 0.74 and 0.80 respectively. For EPQ-PO, the initial EFA resulted in a five-component model that explained 63.0% of the variance. However, an inspection of the scree plot supported a four-component solution (Supplementary file 1). For EPQ-IO, the initial EFA resulted in a four-component model that explained 62.9% of the variance. An inspection of the scree plot supported a four-component solution (Supplementary file 1). An oblique rotation was performed for both EPQ-PO and EPQ-IO. For EPQ-PO, a second EFA with a fixed 4-component solution was conducted in line with the principles described in the analysis of SDS-IO (cf. 3.2). In the second EFA for EPQ-PO, Bartlett's test of sphericity was significant (χ2=478.64, *P* <.001) and KMO was 0.74. The fixed four-component solution explained 56.7% of the variance. An oblique rotation was then conducted for this four-component solution of EPQ-PO. The research team then inspected the four-component solution for both EPQ-PO and EPQ-IO in combination with meaningfulness of the item-factor structure. On this basis, the research group decided to support a four-factor structure. The factor loadings and communalities are presented in Table 2, which shows that even though the number of factors extracted is the same for EPQ-PO and EPQ-IO, the item-factor structure varies between EPQ-PO and EPQ-IO. The pattern matrix and structure matrix of the four-component solution of EPQ-PO and EPQ-IO are presented in Supplementary file 2.

#### 3.3.3 Additional assessment of reliability

After the research team decided how many factors to retain, Cronbach's alpha was conducted for the four factors extracted from EPQ-PO and EPQ-IO, see Table 2.

### 3.4 Psychometric analysis of Student Satisfaction and Self-Confidence in Learning Scale

#### 3.4.1 Reliability

The item analysis of the SCLS is presented in Table 4. The item response frequencies show that most respondents answered *important* or *very important*. Skewness was less than -1 for all 13 items. Kurtosis values were above zero for items 1–7 and 12 and below zero for items 8–11 and 13. Inter-item correlation was above 0.30 for all items except for item S13 (0.13). The low score on item S13 indicated that this item might be removed from the scale. The Cronbach's alpha of the overall SCLS (13 items) was 0.85. However, when an item's alpha score is higher than the total alpha, it is considered advisable to remove the item (Pallant, 2016). After removing item S13 (score 0.88), Cronbach's alpha for SCLS (12 items) was 0.89. Cronbach's alpha of the two subscales (*Satisfaction with Current Learning and Self-Confidence in Learning*) was 0.81 and 0.82, respectively.

**Table 4:** Response-option frequency for SCLS.

Item	SD %	D %	UN %	A %	SA %	Mean ± SD	Item-Total Correlation 13 items (12 items)
S1. The teaching methods used in this simulation were helpful and effective.			1.0	21.9	77.1	4.76 ± 0.450	0.54 (0.53)
S2. The simulation provided me with learning materials and activities to promote my learning the medical surgical curriculum.				24.8	75.2	4.75 ± 0.434	0.55 (0.57)
S3. I enjoyed how my instructor taught the simulation.		1.0	4.8	42.9	51.4	4.45 ± 0.635	0.63 (0.65)
S4. The teaching materials used motivating and helped me to learn.			7.6	24.8	66.7	4.60 ± 0.631	0.71 (0.68)
S5. The way my instructor taught was suitable to the way I learn.		1.0	11.4	34.3	52.4	4.39 ± 0.730	0.69 (0.72)
S6. I am confident that I am mastering the content of the simulation activity that my instructor presented to me.	1.0	3.8	30.5	52.4	12.4	3.71 ± 0.769	0.55 (0.58)
S7. The simulation covered critical content necessary for the mastery.		1.0	6.7	27.6	64.8	4.56 ± 0.664	0.43 (0.45)
S8. I am developing the skills and obtaining the required knowledge to perform in a clinical setting.			6.7	35.2	58.1	4.51 ± 0.622	0.63 (0.60)
S9. My instructors used helpful resources.			1.9	40.0	58.1	4.56 ± 0.536	0.63 (0.65)
S10. It is my responsibility to learn what I need to know from this simulation activity.			3.8	34.3	60.0	4.57 ± 0.571	0.41 (0.43)
S11. I know how to get help when I do not understand the concepts covered in simulation.		1.0	14.3	38.1	45.7	4.30 ± 0.749	0.54 (0.57)
S12. I know how to use simulation activities to learn critical aspects of these skills.		1.0	7.6	44.8	46.7	4.37 ± 0.669	0.66 (0.69)
S13. It is the instructor's responsibility to tell me what I need to learn during class time.	3.8	11.4	41.9	28.6	13.3	3.37 ± 0.986	0.13

SD = Strongly Disagree. D = Disagree. UN = Undecided. A = Agree. SA = Strongly Agree.

#### 3.4.2 Validity

In the following analysis, SCLS was computed including items 1–12, i.e. excluding item S13. Results from Bartlett's tests of sphericity and KMO revealed that the sample met the criteria for conducting factor analyses. Bartlett's test revealed significance (χ2 = 556.07, *P* <.001) and KMO was 0.85. The initial EFA resulted in a two-component model that explained 56.0% of the variance. An inspection of the scree plot suggested a two-factor solution (Supplementary file 1). An oblique rotation was performed. The factor loadings and communalities are presented in Table 2. The research team then inspected the two-component solution for the meaningfulness of the item-factor structure and decided to support a two-factor solution. The pattern matrix and structure matrix are presented in Supplementary file 2.

#### 3.4.3 Additional assessment of reliability

After the research team had concluded on the number of factors to retain, Cronbach's alpha was conducted for the two factors extracted, see Table 2.

## 4 Discussion

In this study we tested three questionnaires, SDS, EPQ and SCLS, for psychometric properties in a Norwegian nursing education context. A summary of the psychometric testing of SDS, EPQ and SCLS is presented in Table 5. SDS-PO and SDS-IO showed a five-factor solution, EPQ-PO and EPQ-IO a four-factor solution and SCLS a two-factor solution. These results supported the number of factors extracted compared to the number of dimensions in the original questionnaires (National League of Nursing, 2020a). In the current study, the number of factors extracted was also in line with results from previous studies, including the American study by Franklin et al. (2014) for SDS-PO and SCLS, the Turkish study by Unver et al. (2017) for SDS-PO and SCLS, the Chinese study by Chan et al. (2015) for SCLS, and the Norwegian study by Tosterud et al. (2014) for SCLS. In our study, all factor loadings had values ≥ 0.40 and the vast majority were > 0.50. (see Table 5). This indicates that the items in the present study strongly influenced their respective factors and therefore, no single item was excluded. As EFA is considered a data reduction method (Kentaro and Yuan, 2010), our results confirmed that the Norwegian versions of SDS, EPQ and SCLS could be reduced to the same number of factors as the number of subscales in the original questionnaires. However, the item-factor structure in our study differed from the original questionnaires’ item-subscales and the item-factor structure found in SDS-PO and SCLS by Franklin et al. (2014), which was in line with the original questionnaires’s subscale structure. The results for SCLS in the present study are in accordance with the Norwegian study by Tosterud et al. (2014) who found no stable item-factor solution for SCLS compared with the original questionnaire’s subscales. However, as both the factor loadings and communalities were acceptable, the item-factor structure was thoroughly investigated by the research team, who were also well versed in SBL. The investigation conclusively supported the item-factor structure. In EFA, it is important that a content validity investigation be undertaken by persons skilled in the subject matter (Furr, 2011). It has been shown that the item-factor structure differs from the item-subscales (National League of Nursing, 2020a) and the item-factor structure in SDS-PO and SCLS, as revealed by Franklin et al. (2014). One explanation for this might be that translating a questionnaire into a foreign language and administering it in a different cultural context could affect the way students interpret and therefore score the items. In the Turkish context, Unver et al. (2017) found that the item-factors differed for one item in SCLS and for several items in SDS-PO compared to the original scales. Similarly, Almeida et al. (2015) in the Portuguese version of SCLS found one item that did not correspond to the factors from the original scales.

**Table 5:** Summary of psychometric testing of SDS, EPQ and SCLS.

Questionnaires	Factor	Items	Cronbach's alpha	Factor loadings (range)
SDS-PO	**1**	D1-D4, D15, D18	0.82	0.45–0.87
	**2**	D5-D9, D11	0.89	−0.42 to −0.89
	**3**	D16, D19, D20	0.69	0.48–0.86
	**4**	D10, D14	0.71	0.62–0.83
	**5**	D12, D13, D17	0.72	0.59–0.88
SDS-IO	**1**	D2-D4, D10, D11	0.78	0.42–0.75
	**2**	D5-D9	0.91	−0.66 to −0.91
	**3**	D19, D20	0.73	0.69–0.78
	**4**	D1, D14-D16, D18	0.76	−0.61 to −0.77
	**5**	D12, D13, D17	0.72	−0.70 to −0.83
EPQ-PO	**1**	E4, E6-E9, E13, E14	0.78	0.41–0.78
	**2**	E1, E11, E12	0.58	−0.53 to −0.89
	**3**	E2, E3, E5	0.66	0.57–0.89
	**4**	E10, E15, E16	0.71	−0.40 to −0.82
EPQ-IO	**1**	E1, E4, E6-E8, E15, E16	0.79	0.46–0.80
	**2**	E2, E3	0.71	0.77–0.81
	**3**	E11, E12	0.79	−0.75 to −0.82
	**4**	E5, E9, E10, E13, E14	0.83	−0.54 to −0.80
SCLS	**1**	S1-S4, S8-S10	0.83	0.49–0.80
	**2**	S5-S7, S11, S12	0.82	−0.64 to −0.80

Our findings show that the Norwegian versions of the questionnaires maintain high internal consistency, with an overall Cronbach's alpha of over 0.80 for each of the three questionnaires. This is in line with the alpha values of the original questionnaires (National League of Nursing, 2020a). However, in SCLS, item S13 was deleted, as this item's alpha showed values above the total for the scale. The decision to remove this item was in line with previous studies (Franklin et al., 2014; Unver et al., 2017). When inspecting item S13 – ‘It is the instructor's responsibility to tell me what I need to learn of the simulation activity content during class time’ – it could be argued that this statement might not reflect an aspect of students’ *satisfaction* or *self-confidence,* as SBL is regarded as an student-active learning approach (Gatewood, 2019). Shifting the responsibility for learning from the student to the instructor might contradict the students’ own perspective of SBL.

In SDS, items D19 and D20 displayed low item-total correlation. These items required the students’ perception of the fidelity of the scenarios and might be challenging for the nursing students in the present study to evaluate as they had limited experience with SBL and the course took place prior to clinical practice. However, despite low item-total correlation, we chose to retain items D19 and D20 for the EFA. According to Tabachnick and Fidell (2019) reporting factors with low reliability can be important when these are regarded as crucial, as fidelity is for SBL (Jeffries and Rogers, 2012). Therefore, the research team decided to retain these items. The respective factor loadings were 0.89 and 0.87 for SDS-PO and 0.78 and 0.69 for SDS-IO, values which further support the contention that these items should be retained in the questionnaire.

In the original SDS and EPQ questionnaires, the layout of the PO and IO are integrated. Based on the results of the current study, we suggest that the SDS and EPQ questionnaires in a Norwegian context should be separated into two parts: one PO and one IO. Separating the PO and IO for SDS and EPQ might increase user flexibility, as it would allow one to easily choose which part students should evaluate, the PO or IO.

A Cronbach's alpha reliability test was conducted for each extracted factor. All factors showed alphas > 0.70 indicating good internal consistency, except for EPQ-PO factor two with a value of 0.58, and factor three with a value of 0.66. Several previous studies (Almeida et al., 2015; Chan et al., 2015; Franklin et al., 2014; Tosterud et al., 2014; Unver et al., 2017) refer to the same item-factor structure as in the original item-subscale structure (National League of Nursing, 2020a). The present study proposes that the item-factor structure found in this Norwegian study has acceptable internal consistency.

### 4.1 Implications for nursing education

The Norwegian versions of the SDS, EPQ and SCLS could be valuable instruments for nurse educators for the development, implementation and evaluation of SBL in Norway. Adding these scales to the instruments available in Norwegian contributes to the sharing of common international values of simulation, the formation of a mutual dialogue, and the possibility of research comparing simulation effectiveness between countries and languages, as pointed out by Kardong-Edgren et al. (2010).

The SDS, EPQ and SCLS questionnaires can be used to assess simulation design, educational practice and satisfaction with instruction, and self-confidence in SBL. Investigations of nursing students’ perception and evaluation of SBL might contribute to improving educational practices.

## 5 Limitations

The sample size of the study may be considered small. Because the study included a cohort of nursing students in a university, it was difficult to increase the sample size. However, as discussed earlier, there is no clear consensus as to what constitutes a large enough sample size (Bujang et al., 2012; Watkins, 2018). Literature on EFA claims that a sample size of 100 is acceptable when the variables are strong, that is with factor loadings > .80 and communalities > 0.50 (Watkins, 2018). Most of the communalities in our study were > 0.50 and most of factor loadings were > 0.70. The study was conducted in a cohort at a single university. A multi-site study could increase the transferability; however, given the stance that the SBL should be comparable in terms of what is being evaluated, the multi-site perspective was not possible for this study. Following EFA, the next step should be to conduct a confirmatory factor analysis (CFA). We recommend that this be done in a new sample (Kentaro and Yuan, 2010).

## 6 Conclusion

The present study has provided empirical evidence to support the construct validity and reliability of the Norwegian versions of the SDS, EPQ and SCLS for the evaluation of SBL in nursing education. The three questionnaires showed acceptable internal consistency and the number of factors extracted was in line with the original number of subscales. However, the item-factor structure differed from the original item-subscales. The Norwegian versions of the SDS, EPQ and SCLS are easy for faculty to administer and can be used by nursing students to evaluate important aspects of SBL, although they necessitate a different item-factor structure compared to the original work. Further multi-site studies are needed to perform a CFA in a large new sample.

## Funding

This research did not receive any specific grant from funding agencies in the public, commercial, or not-for-profit sectors.

## Declaration of Competing Interest

None.

## Appendix A Supplementary materials

Application

## Appendix B Supplementary materials

Application

## Footnotes

Supplementary data associated with this article can be found, in the online version, at 10.1016/j.ijnsa.2020.100012.

## References

[bib0001] Adamson K.A., Kardong-Edgren S., Willhaus J. (2013). An updated review of published simulation evaluation instruments. Clin. Simul. Nurs..

[bib0002] Aebersold M. (2018). Simulation-based learning: no longer a novelty in undergraduate education. OJIN: Online J. Issues Nurs..

[bib0003] Al Sabei S.D., Lasater K. (2016). Simulation debriefing for clinical judgment development: a concept analysis. Nurse Educ. Today.

[bib0004] Almeida R.G., Jorge B.M., Souza-Junior V.D., Mazzo A., Martins J.C.A., Negri E.C., Mendes I.A.C. (2018). Trends in research on simulation in the teaching of nursing: an integrative review. Nurs. Educ. Perspect..

[bib0005] Almeida R.G., Mazzo A., Martins J.C., Baptista R.C., Girao F.B., Mendes I.A. (2015). Validation to Portuguese of the scale of student satisfaction and self-confidence in learning. Rev. Lationo-Am. Enfermagem.

[bib0006] Bujang M.A., Ab Ghani P., Soelar S.A., Zulkifli N.A. (2012). 2012 International Conference on Statistics in Science, Business and Engineering (ICSSBE).

[bib0007] Cant R.P., Cooper S.J. (2017). Use of simulation-based learning in undergraduate nurse education: an umbrella systematic review. Nurse Educ. Today.

[bib0008] Chan J.C.K., Fong D.Y.T., Tang J.J., Pui Gay K., Hui J. (2015). The Chinese student satisfaction and self-confidence scale is reliable and valid. Clin. Simul. Nurs..

[bib0009] de Winter J.C.F., Dodou D., Wieringa P.A. (2009). Exploratory factor analysis with small sample sizes. Multivar. Behav. Res..

[bib0010] Doolen J., Mariani B., Atz T., Horsley T.L., Rourke J.O., McAfee K., Cross C.L. (2016). High-fidelity simulation in undergraduate nursing education: a review of simulation reviews. Clin. Simul. Nurs..

[bib0011] Field A. (2009).

[bib0012] Franklin A.E., Burns P., Lee C.S. (2014). Psychometric testing on the NLN student satisfaction and self-confidence in learning, simulation design scale, and educational practices questionnaire using a sample of pre-licensure novice nurses. Nurse Educ. Today.

[bib0013] Furr M.R. (2011). Scale Construction and Psychometrics for Social and Personality Psychology.

[bib0015] Gatewood E. (2019). Use of simulation to increase self-directed learning for nurse practitioner students. J. Nurs. Educ..

[bib0016] Haddeland K., Slettebø Å., Carstens P., Fossum M. (2018). Nursing students managing deteriorating patients: a systematic review and meta-analysis. Clin. Simul. Nurs..

[bib0017] Hair J.F., Black W.C., Babin B.j., Anderson R.E. (2014).

[bib0018] Haukedal T.A., Reierson I.Å., Hedeman H., Bjørk I.T. (2018). The impact of a new pedagogical intervention on nursing students’ knowledge acquisition in simulation-based learning: a quasi-experimental study. Nurs. Res. Pract..

[bib0019] Hayden J., Smiley R.A., Alexander M., Kardong-Edgren S., Jeffries P.R. (2014). The NCSBN national simulation study: a longitudinal, randomized, controlled study replacing clinical hours with simulation in prelicensure nursing education. J. Nurs. Regul..

[bib0020] Hogarty K.Y., Hines C.V., Kromrey J.D., Ferron J.M., Mumford K.R. (2005). The quality of factor solutions in exploratory factor analysis: the influence of sample size, communality, and over determination. Educ. Psychol. Meas..

[bib0021] IBM Corp., 2019. IBM SPSS statistics for windows, version 26. Available at https://www.ibm.com/products/spss-statistics .

[bib0022] INACSL Standards Committee (2016). INACSL standards of best practice: simulation^SM^ simulation design. Clin. Simul. Nurs..

[bib0023] Jeffries P.R. (2005). A framework for designing, implementing, and evaluating simulations used as teaching strategies in nursing. Nurs. Educ. Perspect..

[bib0024] Jeffries P.R., Rizzolo M.A. (2006).

[bib0025] Jeffries P.R., Rogers K.J., Jeffries P.R. (2012). Simulation in Nursing Education: From Conceptualization to Evaluation.

[bib0026] Jeppesen K.H., Christiansen S., Frederiksen K. (2017). Education of student nurses - a systematic literature review. Nurse Educ. Today.

[bib0027] Kardong-Edgren S., Adamson K.A., Fitzgerald C. (2010). A review of currently published evaluation instruments for human patient simulation. Clin. Simul. Nurs..

[bib0028] Kentaro H., Yuan K.-H., Salkind J.N. (2010). Encyclopedia of Research Design.

[bib0029] Kirkpatrick D.L., Ely D.P., Plomp T. (1996). Classic Writings on Instructional Technology (119-142).

[bib0030] Lioce L., Lopreiato J., Downing D., Chang T., Robertson J., Anderson M., Diaz D., Spain A. (2020). https://www.ssih.org/dictionary.

[bib0031] Lovett S., Roche J., Hunter S., Symonds I., Tomlinson N., Gagnon R., Charlin B., Mattes J.J.M. (2016). Respective value of the traditional clinical rotation and high fidelity simulation on the acquisition of clinical reasoning skills in medical students – a randomized controlled trial. MedEdPublish.

[bib0032] National League of Nursing, 2020a. Descriptions of available instruments. http://www.nln.org/professional-development-programs/research/tools-and-instruments/descriptions-of-available-instruments Access date 20200511.

[bib0033] National League of Nursing, 2020b. Use of NLN surveys and research instruments. http://www.nln.org/professional-development-programs/research/tools-and-instruments Access date 20200511.

[bib0034] Osborne J. (2014).

[bib0035] Pallant J. (2016).

[bib0036] Patton M.Q. (2015).

[bib0037] Pett M.A., Lackey N.R., Sullivan J.J. (2003).

[bib0038] Polit D.F., Beck C.T. (2018).

[bib0039] Tabachnick B.G., Fidell L.S. (2019).

[bib0040] Thim T., Krarup N.H.V., Grove E.L., Rohde C.V., Løfgren B. (2012). Initial assessment and treatment with the Airway, Breathing, Circulation, Disability, Exposure (ABCDE) approach. Int. J. Gen. Med..

[bib0041] Tosterud R. (2015).

[bib0042] Tosterud R., Petzäll K., Hedelin B., Hall-Lord M.L. (2014). Psychometric testing of the Norwegian version of the questionnaire, student satisfaction and self-confidence in learning, used in simulation. Nurse Educ. Pract..

[bib0043] Unver V., Basak T., Watts P., Gaioso V., Moss J., Tastan S., Iyigun E., Tosun N. (2017). The reliability and validity of three questionnaires: the student satisfaction and self-confidence in learning scale, simulation design scale, and educational practices questionnaire. Contemp. Nurse.

[bib0044] Warren J.N., Luctkar-Flude M., Godfrey C., Lukewich J. (2016). A systematic review of the effectiveness of simulation-based education on satisfaction and learning outcomes in nurse practitioner programs. Nurse Educ. Today.

[bib0045] Watkins M.W. (2018). Exploratory factor analysis: a guide to best practice. J. Black Psychol..

[bib0046] World Medical Association (2001). World medical association declaration of Helsinki. Ethical principles for medical research involving human subjects. Bull. World Health Org..

