# Supplementary material for: Psychometric testing of the Norwegian version of the Simulation Design Scale, the Educational Practices Questionnaire and the Student Satisfaction and Self-Confidence in Learning Scale in nursing education
Source: Int J Nurs Stud Adv. 2020 Oct 25;2:100012. doi: 10.1016/j.ijnsa.2020.100012 (PMC11080358; doi:10.1016/j.ijnsa.2020.100012)
Supplement: Application [file mmc1.pdf]

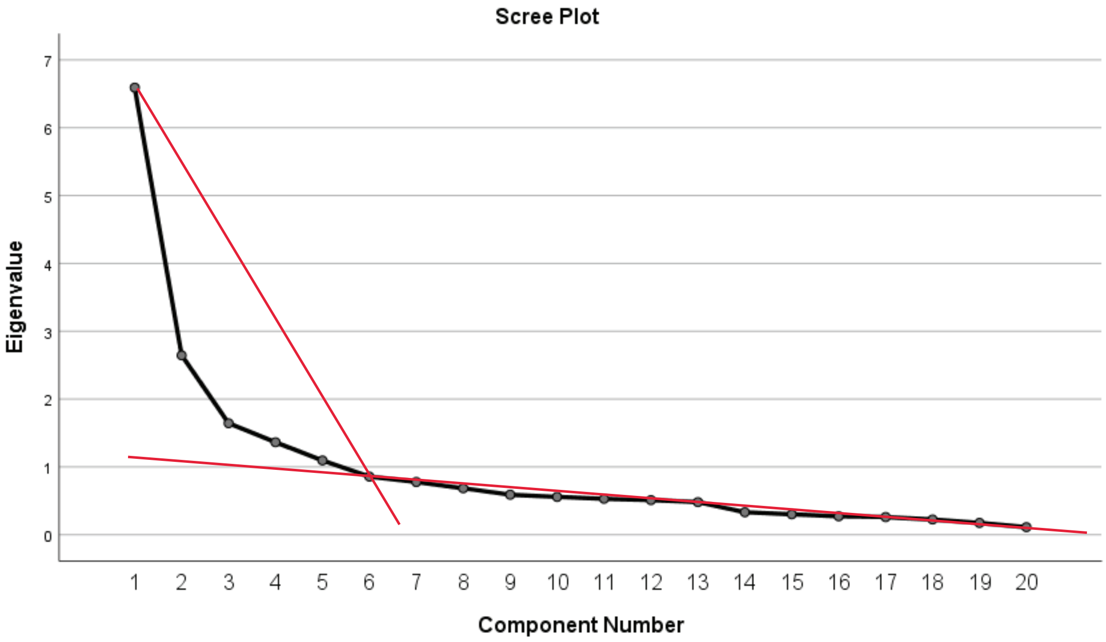

Supplementary file 1. Scree plot SDS-PO

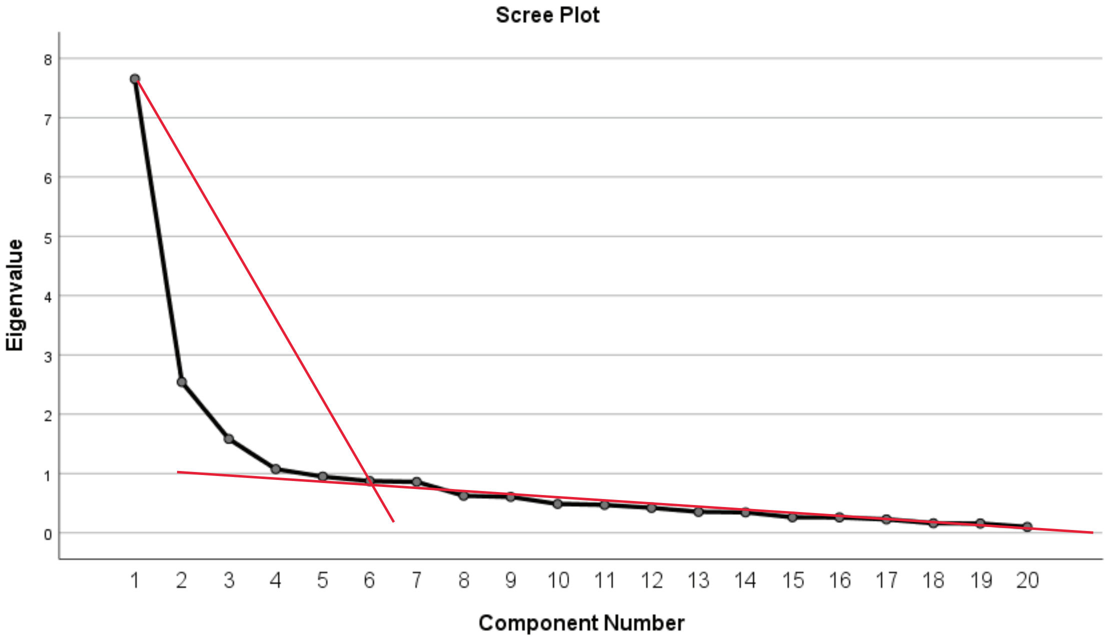

Supplementary file 1. Scree plot SDS-IO

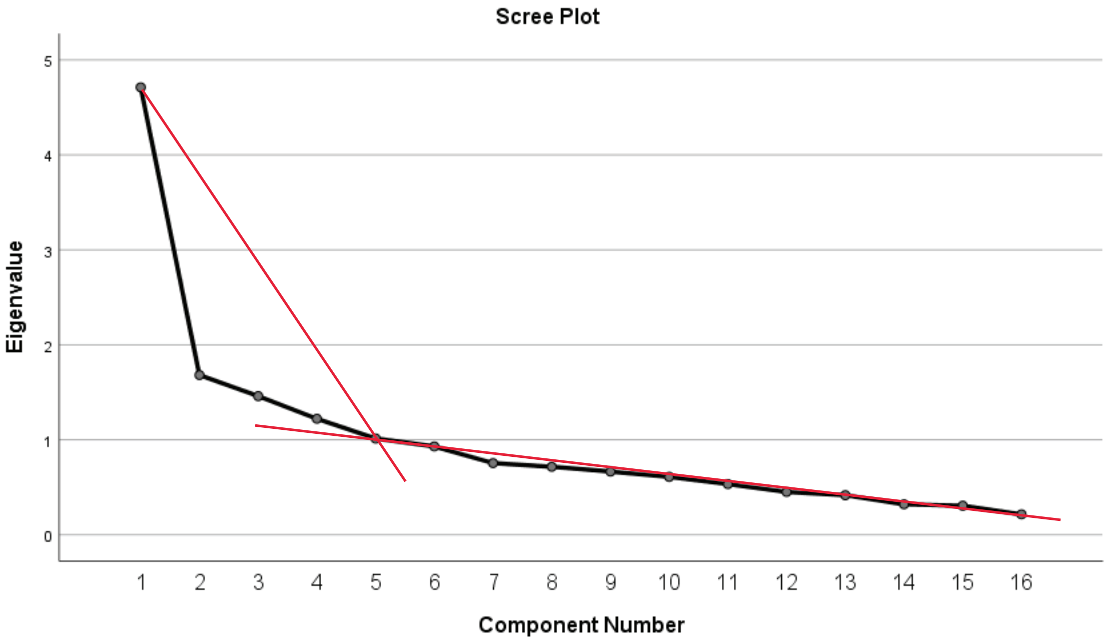

Supplementary file 1. Scree plot EPQ-PO

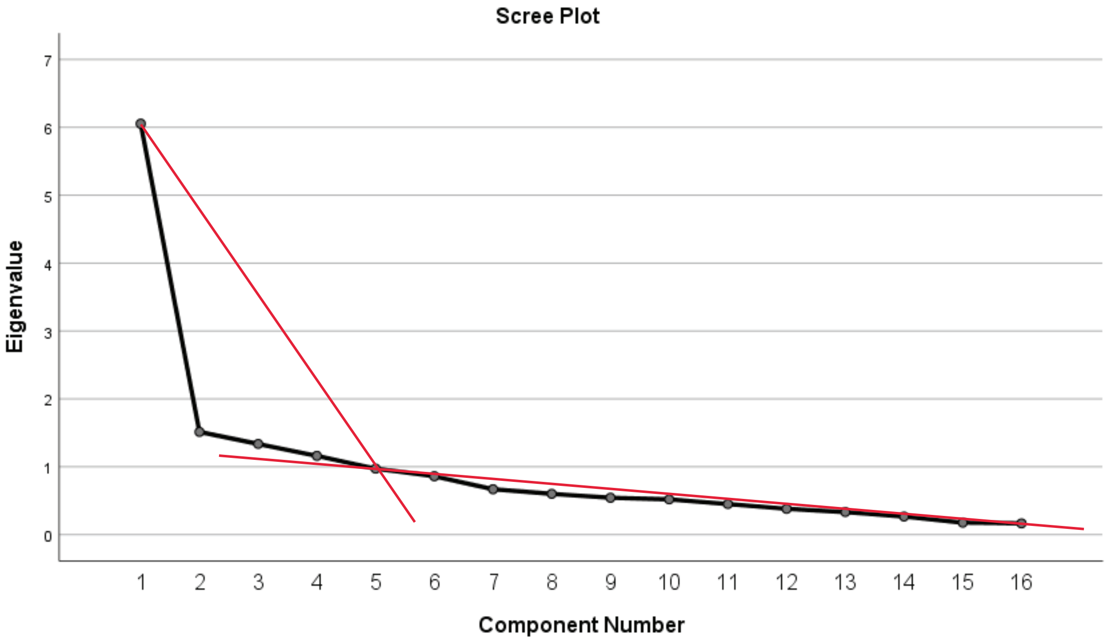

Supplementary file 1. Scree plot EPQ-IO

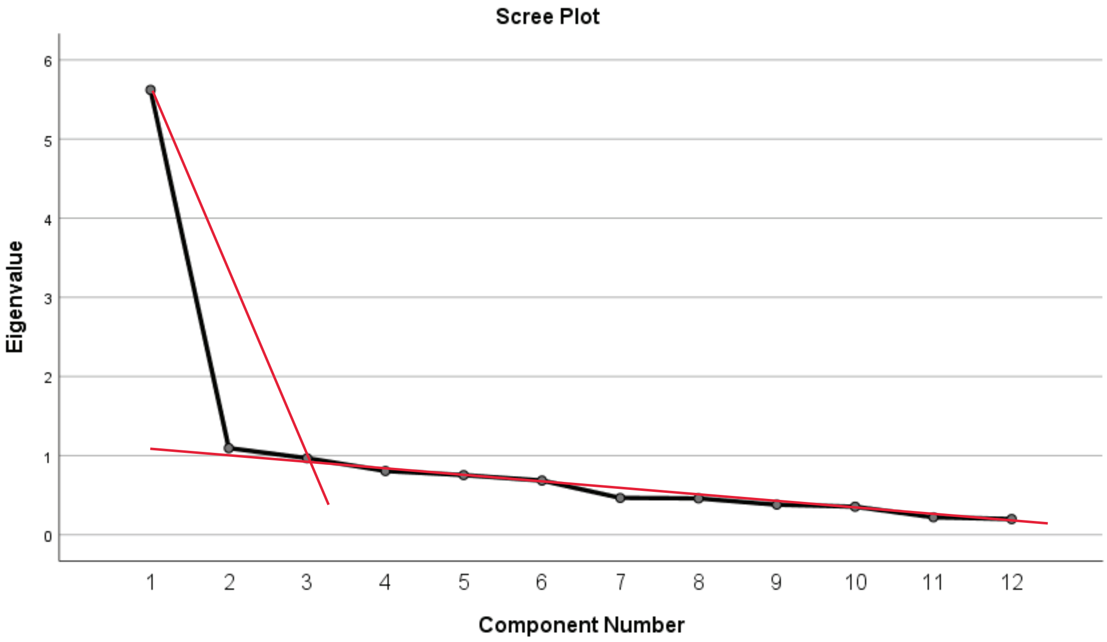

Supplementary file 1. Scree plot SCLS
