# Supplementary material for: Psychometric testing of the Norwegian version of the Simulation Design Scale, the Educational Practices Questionnaire and the Student Satisfaction and Self-Confidence in Learning Scale in nursing education
Source: Int J Nurs Stud Adv. 2020 Oct 25;2:100012. doi: 10.1016/j.ijnsa.2020.100012 (PMC11080358; doi:10.1016/j.ijnsa.2020.100012)
Supplement: Application [file mmc2.docx]

|  | | | | | | | | | | | |
| --- | --- | --- | --- | --- | --- | --- | --- | --- | --- | --- | --- |
| Pattern matrix | | | | | | Structure matrix | | | | | |
| Item | Component | | | | | Item | Component | | | | |
|  | 1 | 2 | 3 | 4 | 5 |  | 1 | 2 | 3 | 4 | 5 |
| D1 | **0.869** |  |  |  |  | D2 | **0.840** | -0.346 |  |  | 0.437 |
| D2 | **0.763** |  |  |  |  | D1 | **0.786** |  |  |  |  |
| D18 | **0.604** |  |  |  |  | D18 | **0.686** |  | 0.367 |  | 0.402 |
| D3 | **0.574** |  |  |  |  | D3 | **0.686** | -0.410 |  |  | 0.469 |
| D4 | **0.573** |  |  |  |  | D4 | **0.682** | -0.344 |  | 0.336 | 0.471 |
| D15 | **0.446** |  | 0.397 |  |  | D15 | **0.632** | -0.315 | 0.538 |  | 0.476 |
| D6 |  | **-0.889** |  |  |  | D6 |  | **-0.867** |  |  |  |
| D8 |  | **-0.861** |  |  |  | D9 |  | **-0.843** |  | 0.336 |  |
| D7 |  | **-0.831** |  |  |  | D7 | 0.344 | **-0.838** |  |  |  |
| D9 |  | **-0.830** |  |  |  | D8 |  | **-0.836** |  |  |  |
| D5 |  | **-0.742** |  |  |  | D5 | 0.302 | **-0.738** |  |  |  |
| D11 |  | **-0.415** |  | 0.407 |  | D11 | 0.334 | **-0.578** |  | 0.523 | 0.405 |
| D19 |  |  | **0.859** |  |  | D20 |  |  | **0.857** |  |  |
| D20 |  |  | **0.856** |  |  | D19 |  |  | **0.847** |  |  |
| D16 |  |  | **0.483** | -0.320 |  | D16 | 0.497 | -0.394 | **0.594** |  | 0.459 |
| D10 |  |  |  | **0.828** |  | D10 |  |  |  | **0.836** |  |
| D14 |  |  |  | **0.616** | 0.333 | D14 |  | -0.371 |  | **0.679** | 0.429 |
| D17 |  |  |  |  | **0.881** | D17 |  |  |  |  | **0.831** |
| D12 |  |  |  |  | **0.666** | D12 | 0.488 |  |  |  | **0.749** |
| D13 |  |  |  |  | **0.591** | D13 | 0.511 |  |  |  | **0.706** |

Extraction Method: Principal Component Analysis.

Rotation Method: Oblimin with Kaiser Normalization. Rotation converged in 9 iterations.

**Supplementary file 2. SDS-PO pattern matrix and structure matrix**

|  | | | | | | | | | | | | |
| --- | --- | --- | --- | --- | --- | --- | --- | --- | --- | --- | --- | --- |
| Pattern Matrix | | | | | | Structure Matrix | | | | | |  |
| Item | Component | | | | | Item | Component | | | | |  |
|  | 1 | 2 | 3 | 4 | 5 |  | 1 | 2 | 3 | 4 | 5 |  |
| D3 | **0.749** |  |  |  |  | D2 | **0.793** | -0.459 |  | -0.337 |  |  |
| D2 | **0.733** |  |  |  |  | D3 | **0.789** |  |  | -0.411 | -0.420 |  |
| D10 | **0.563** |  |  |  |  | D4 | **0.663** | -0.514 |  | -0.426 | -0.311 |  |
| D4 | **0.501** |  |  |  |  | D10 | **0.640** |  |  | -0.326 | -0.393 |  |
| D9 |  | **-0.911** |  |  |  | D11 | **0.609** | -0.468 | -0.362 | -0.485 |  |  |
| D6 |  | **-0.893** |  |  |  | D9 | 0.310 | **-0.893** |  | -0.335 |  |  |
| D7 |  | **-0.861** |  |  |  | D7 | 0.379 | **-0.887** |  | -0.383 |  |  |
| D8 |  | **-0.815** |  |  |  | D6 | 0.327 | **-0.882** |  | -0.326 |  |  |
| D5 |  | **-0.663** |  |  |  | D8 | 0.465 | **-0.865** |  | -0.326 |  |  |
| D19 |  |  | **0.779** |  |  | D5 | 0.500 | **-0.735** |  |  |  |  |
| D20 |  |  | **0.686** |  |  | D19 |  |  | **0.810** |  |  |  |
| D11 | 0.417 |  | **-0.421** |  |  | D20 | 0.301 |  | **0.729** | -0.430 | -0.315 |  |
| D16 |  |  |  | **-0.766** |  | D16 | 0.418 | -0.309 |  | **-0.823** | -0.378 |  |
| D18 |  |  | 0.352 | **-0.664** |  | D18 | 0.329 | -0.360 | 0.467 | **-0.797** | -0.523 |  |
| D1 |  |  |  | **-0.644** |  | D1 | 0.485 | -0.442 |  | **-0.737** |  |  |
| D14 |  |  | -0.473 | **-0.619** |  | D15 | 0.329 |  | 0.398 | **-0.729** | -0.552 |  |
| D15 |  |  |  | **-0.610** |  | D14 | 0.320 | -0.338 | -0.371 | **-0.650** | -0.326 |  |
| D13 |  |  |  |  | **-0.834** | D13 |  | -0.306 |  | -0.389 | **-0.858** |  |
| D17 |  |  |  |  | **-0.818** | D17 | 0.371 |  |  | -0.395 | **-0.842** |  |
| D12 | 0.323 |  |  |  | **-0.703** | D12 | 0.555 |  |  | -0.391 | **-0.807** |  |

Extraction Method: Principal Component Analysis.

Rotation Method: Oblimin with Kaiser Normalization. Rotation converged in 13 iterations.

**Supplementary file 2. SDS-IO pattern matrix and structure matrix**

|  | | | | | | | | | |
| --- | --- | --- | --- | --- | --- | --- | --- | --- | --- |
| Pattern Matrix | | | | | Structure Matrix | | | | |
| Item | Component | | | | Item | Component | | | |
|  | 1 | 2 | 3 | 4 |  | 1 | 2 | 3 | 4 |
| E6 | **0.783** |  |  |  | E9 | **0.735** |  |  | -0.326 |
| E9 | **0.707** |  |  |  | E7 | **0.720** |  |  | -0.394 |
| E7 | **0.690** |  |  |  | E6 | **0.697** |  |  |  |
| E8 | **0.573** |  |  |  | E8 | **0.656** | -0.391 | 0.312 |  |
| E4 | **0.518** |  | 0.367 |  | E4 | **0.627** |  | 0.510 |  |
| E14 | **0.490** |  |  |  | E14 | **0.558** |  |  |  |
| E13 | **0.413** |  |  |  | E13 | **0.515** | -0.416 |  | -0.308 |
| E11 |  | **-0.885** |  |  | E11 |  | **-0.847** |  |  |
| E12 |  | **-0.759** |  |  | E12 |  | **-0.773** |  | -0.352 |
| E1 |  | **-0.528** |  |  | E1 | 0.324 | **-0.543** |  |  |
| E3 |  |  | **0.885** |  | E3 |  |  | **0.854** |  |
| E2 |  |  | **0.790** |  | E2 |  |  | **0.775** |  |
| E5 |  | -0.319 | **0.567** |  | E5 |  | -0.447 | **0.646** | -0.378 |
| E15 |  |  |  | **-0.821** | E15 | 0.335 |  |  | **-0.851** |
| E16 |  |  |  | **-0.744** | E16 | 0.344 |  |  | **-0.789** |
| E10 |  | -0.349 |  | **-0.401** | E10 | 0.326 | -0.493 | 0.366 | **-0.534** |

Extraction Method: Principal Component Analysis.

Rotation Method: Oblimin with Kaiser Normalization. Rotation converged in 10 iterations.

**Supplementary file 2. EPQ-PO pattern matrix and structure matrix**

|  | | | | | | | | | | |
| --- | --- | --- | --- | --- | --- | --- | --- | --- | --- | --- |
| Pattern Matrix | | | | | Structure Matrix | | | | |  |
| Item | Component | | | | Item | Component | | | |  |
|  | 1 | 2 | 3 | 4 |  | 1 | 2 | 3 | 4 |  |
| E7 | **0.798** |  |  |  | E7 | **0.781** |  |  | -0.314 |  |
| E15 | **0.722** |  |  |  | E15 | **0.780** |  |  | -0.431 |  |
| E4 | **0.662** |  |  |  | E4 | **0.706** | 0.343 |  | -0.354 |  |
| E6 | **0.576** |  |  | -0.361 | E8 | **0.672** | 0.404 | -0.397 | -0.407 |  |
| E16 | **0.541** |  | -0.317 |  | E16 | **0.645** | 0.357 | -0.473 | -0.303 |  |
| E8 | **0.534** |  |  |  | E6 | **0.601** |  |  | -0.490 |  |
| E1 | **0.457** |  |  |  | E1 | **0.455** |  |  |  |  |
| E3 |  | **0.807** |  |  | E3 |  | **0.823** |  |  |  |
| E2 |  | **0.773** |  |  | E2 | 0.311 | **0.820** |  |  |  |
| E12 |  |  | **-0.823** |  | E12 |  |  | **-0.862** | -0.325 |  |
| E11 |  |  | **-0.749** |  | E11 | 0.408 |  | **-0.812** | -0.333 |  |
| E13 |  |  |  | **-0.801** | E13 | 0.446 |  | -0.334 | **-0.856** |  |
| E14 |  |  | -0.402 | **-0.722** | E14 | 0.318 |  | -0.543 | **-0.776** |  |
| E5 |  | 0.362 |  | **-0.687** | E5 | 0.427 | 0.480 |  | **-0.755** |  |
| E9 |  |  |  | **-0.569** | E9 | 0.525 |  | -0.371 | **-0.702** |  |
| E10 |  | 0.490 |  | **-0.540** | E10 |  | 0.582 |  | **-0.611** |  |

Extraction Method: Principal Component Analysis.

Rotation Method: Oblimin with Kaiser Normalization. Rotation converged in 11 iterations.

**Supplementary file 2. EPQ-IO pattern matrix and structure matrix**

|  | | | | | | |
| --- | --- | --- | --- | --- | --- | --- |
| Pattern Matrix | | | Structure Matrix | | |  |
| Item | Component | | Item | Component | |  |
|  | 1 | 2 |  | 1 | 2 |  |
| S9 | **0.799** |  | S9 | **0.818** | -0.499 |  |
| S2 | **0.784** |  | S4 | **0.773** | -0.514 |  |
| S1 | **0.735** |  | S2 | **0.756** | -0.408 |  |
| S4 | **0.717** |  | S1 | **0.725** | -0.412 |  |
| S10 | **0.640** |  | S3 | **0.691** | -0.636 |  |
| S8 | **0.527** |  | S8 | **0.672** | -0.556 |  |
| S3 | **0.485** | -0.353 | S10 | **0.592** |  |  |
| S12 |  | **-0.797** | S12 | 0.522 | **-0.830** |  |
| S11 |  | **-0.796** | S5 | 0.627 | **-0.789** |  |
| S7 |  | **-0.714** | S11 | 0.419 | **-0.770** |  |
| S6 |  | **-0.692** | S6 | 0.466 | **-0.729** |  |
| S5 |  | **-0.642** | S7 | 0.334 | **-0.666** |  |

Extraction Method: Principal Component Analysis.

Rotation Method: Oblimin with Kaiser Normalization. Rotation converged in 6 iterations.

**Supplementary file 2. SCLS pattern matrix and structure matrix**
